# Supplementary material for: Not All Workers Experience Equal Sleep Changes: Insights from the “WorkInCovid” Project
Source: Clocks Sleep. 2025 Mar 10;7(1):13. doi: 10.3390/clockssleep7010013 (PMC11941416; doi:10.3390/clockssleep7010013)
Supplement: Supplementary file 1 [file clockssleep-07-00013-s001.zip › clockssleep-3441341-supplementary.pdf]

SUPPLEMENT 1

Table S1. PSQI components before and during WH in the whole sample. Contingency table with number of subjects in the whole sample for each PSQI components' score (range 0-3, with higher scores implying greater difficulties), before WH (pre-WH) and during WH (n=737).

						p value[1]
		**Subjective sleep quality**				
		**WH**				
		**0**	**1**	**2-3**	**Total (%)**	
	**0**	70	24	4	13.3	
**Pre-WH**	**1**	45	365	53	62.8	0.02
	**2-3**	7	63	106	23.9	
	**Total (%)**	16.6	61.3	22.1		
		**Sleep latency**				
		**WH**				
		**0**	**1**	**2-3**	**Total (%)**	
	**0**	199	74	8	38.1	
**Pre-WH**	**1**	43	329	36	55.4	0.0002
	**2-3**	4	15	29	6.5	
	**Total (%)**	33.4	56.7	9.9		
		**Sleep duration**				
		**WH**				
		**0**	**1**	**2-3**	**Total (%)**	
	**0**	140	29	14	25.0	
**Pre-WH**	**1**	93	168	42	41.2	≪0.001
	**2-3**	20	86	143	33.8	
	**Total (%)**	34.3	38.4	27.3		
		**Sleep efficiency**				
		**WH**				
		**0**	**1**	**2-3**	**Total (%)**	
	**0**	441	69	25	72.6	
**Pre-WH**	**1**	73	51	17	19.1	0.06
	**2-3**	9	14	38	8.3	
	**Total (%)**	70.9	18.2	10.9		
		**Sleep disturbances**				
		**WH**				
			**0-1**[2]	**2-3**	**Total (%)**	
**Pre-WH**	**0-1**[2]		546	632	81.3	≪0.001
	**2-3**		31	107	18.7	
	**Total (%)**		77.0	23.0		
		**Use of sleep medications**				
		**WH**				
		**0**	**1**	**2-3**	**Total (%)**	
	**0**	615	14	14	87.2	
**Pre-WH**	**1**	13	27	8	6.5	0.31
	**2-3**	8	5	33	6.2	
	**Total (%)**	86.3	6.2	7.5		
		**Daytime dysfunction**				
		WH				
		**0**	**1**	**2-3**	**Total (%)**	
	**0**	428	19	33	65.1	
**Pre-WH**	**1**	20	49	24	12.6	0.004
	**2-3**	72	13	79	22.3	
	**Total (%)**	70.6	11.0	18.4		

[1] Stuart-Maxwell test. Numbers of subjects with score 2 and 3 were grouped due to the low number of subjects in individual scores.

[2] Numbers of subjects with score 0 and 1 were grouped due to the low number of subjects in individual scores.

SUPPLEMENT 2

Table S2. Association of overall sleep quality (total PSQI score) with sociodemographic and work-related characteristics of the participants. Chi-square test for categorical variable, Wilcoxon test or Kruskal-Wallis test with Bonferroni correction for continuous variables. Very low frequencies have been aggregated with those of neighboring classes for both statistical (chi-squared test) and regulatory reasons, where possible. Otherwise, the low frequencies were not reported, in compliance with the Italian legislation on the protection of personal data (Deontological rules for processing for statistical or scientific research purposes carried out within the National Statistical System, resolution n. 514/2018). For the sake of comparison, these aggregations were made in the same way for all the other confounding variables, regardless of the actual number in the specific cells.

**PSQI[1,2]**	***GENDER***				
	**Men**	**Women**	***p*-value**		
Pre-pandemic	3 (1, 5)	4 (2, 6)	<<0.001		
During WH	3 (1, 5)	4 (2, 6)	<<0.001		
Change during WH compared with pre-WH	-2 (-4, 0)	-1 (-3, 1)	0.41		
	AGE				
	**≤39 ys**	**40-49**	**50-59**	**≥60 ys**	***p*-value**
Pre-pandemic	5 (3,6)	5 (4,6)	5 (4,7)	5 (4,7)	0.24
During WH	5 (3,6)	5 (3,7)	5 (3,7)	5 (4,7)	0.64
Change during WH compared with pre-WH	0 (-1, 1)	0 (-1, 1)	0 (-1, 1)	0 (-1, 1)	0.81
	LIVING ALONE OR NOT DURING THE PANDEMIC				
	**Not alone**	**Alone**	***p*-value**		
Pre-pandemic	5 (4, 6)	5 (4, 7)	0.54		
During WH	5 (3, 6)	5 (3, 7)	0.35		
Change during WH compared with pre-WH	0 (-1, 1)	0 (-1, 1)	0.03[3]		
	PROFESSIONAL PROFILE				
	**Technical staff**	**Administrat. staff**	**Technologist**	**Researcher**	***p*-value**
Pre-pandemic	5 (4, 7)	6 (5, 7)	5 (4, 7)	5 (3, 6)	<<0.001[4]
During WH	5 (4, 7)	5 (4, 8)	5 (4, 7)	4 (3, 6)	0.004[4]
Change during WH compared with pre-WH	0 (-1, 1)	0 (-1, 1)	0 (-1, 1)	0 (-1, 1)	0.70
	CAREGIVING TASKS FOR COHABITING PEOPLE				
	**No**	**Yes**	***p*-value**		
Pre-pandemic	5 (4, 6)	5 (4, 7)	0.48		

During WH	5 (3,7)	4 (3, 5.5)	0.05		
Change during WH compared with pre-WH	0 (-1, 1)	0 (-2, 1)	0.03[3]		
	CAREGIVING TASKS FOR NON-COHABITING PEOPLE				
	**No**	**Yes**	***p*-value**		
Pre-pandemic	3 (2, 5)	4 (2, 6)	0.02[3]		
During WH	5 (3, 6)	5 (4, 7)	0.02[3]		
Change during WH compared with pre-WH	0 (-1, 1)	0 (-1, 1)	0.63		
	MACRO-REGION				
	**North**	**Centre**	**South**	**Islands**	***p*-value**
Pre-pandemic	5 (4, 6)	5 (4, 7)	5 (4, 6)	5 (4, 6)	0.90
During WH	5 (3, 6)	5 (3, 7)	5 (3, 6)	5 (3, 6)	0.84
Change during WH compared with pre-WH	0 (-1, 1)	0 (-1, 1)	0 (-1, 1)	0 (-1, 1)	0.98
	HOME-WORK COMMUTING TIME				
	**<15 min**	**15-30 min.**	**30-60 min.**	**>60 min**	***p*-value**
Pre-pandemic	5 (4, 6)	5 (3, 6)	5 (4, 7)	5 (4, 7)	0.18
During WH	5 (3, 7)	5 (3, 6)	5 (4, 7)	5 (3, 6)	0.39
Change during WH compared with pre-WH	0 (-1, 1)	0 (-1, 1)	0 (-1, 1)	-1 (-1, 1)	0.12
	TYPE OF WORKROOM AT HOME				
	Different rooms, with circumstances	**Fixed and already used as a studio**	**Fixed and temporarily used as a studio**	**Fixed but not used as a studio**	***p*-value**
Pre-pandemic	5 (4, 6)	5 (3.5, 7)	5 (4, 6)	5 (4, 6.5)	0.63
During WH	5 (4, 6)	5 (3, 7)	5 (3, 6.5)	5 (3, 6.5)	0.90
Change during WH compared with pre-WH	0 (-1, 1)	0 (-1, 1)	0 (-1, 1)	0 (-1, 0)	0.11
	FREQUENCY OF SHARING OF THE WORKROOM AT HOME				
	**Never**	**Occasionally**	**Often or always**	***p*-value**	
Pre-pandemic	5 (4, 7)	5 (4, 6)	5 (4, 6)	0.48	
During WH	5 (3, 7)	5 (3, 6)	5 (4, 7)	0.44	
Change during WH compared with pre-WH	0 (-1, 1)	0 (-1, 1)	0 (-1, 1)	0.05[5]	

[1] Median and inter-quartile interval.

[2] 737 respondents.
[3] The estimated difference is negligible.
[4] The total score of Researchers is significantly lower than the total score of both Technical and Administrative staff. The estimated difference is of -1.
[5] All the pairwise comparisons are not significant.

SUPPLEMENT 3

Table S3. PSQI components before and during WH in poor and good sleepers. Contingency table with number of subjects in the poor sleeper group (n=285) and the good sleeper group (n=452) for each components' score (range 0-3, with higher scores implying greater difficulties), before WH (pre-WH) and during WH. The p-values refer to the McNemar test. Score aggregation was used to handle low counts.

		**Subjective sleep quality**			***p-value***
		Poor sleepers			
		**WH**			
		**0-1**	**2-3**	**Total (%)**	
	**0-1**	122	20	49.8	<0.001
**Pre-WH**	**2-3**	57	86	50.2	
	**Total (%)**	62.8	37.2		
		Good sleepers			
		**WH**			
		**0-1**	**2-3**	**Total (%)**	
	**0-1**	382	37	92.7	0.001
**Pre-WH**	**2-3**	13	20	7.3	
	**Total (%)**	87.4	12.6		
		**Sleep latency**			
		Poor sleepers			
		**WH**			
		**0-1**	**2-3**	**Total (%)**	
	**0-1**	217	21	83.5	0.75
**Pre-WH**	**2-3**	18	29	16.5	
	**Total (%)**	82.5	17.5		
		Good sleepers			
		**WH**			
		**0-1**	**2-3**	**Total (%)**	
	**0-1**				<0.001[1]
**Pre-WH**	**2-3**				
	**Total (%)**				
		**Sleep duration**			
		Poor sleepers			
		**WH**			
		**0-1**	**2-3**	**Total (%)**	
	**0-1**	86	16	35.8	≪0.001
**Pre-WH**	**2-3**	69	114	64.2	
	**Total (%)**	54.4	45.6		
		Good sleepers			
		**WH**			
		**0-1**	**2-3**	**Total (%)**	
	**0-1**	344	42	85.4	0.65
**Pre-WH**	**2-3**	37	29	14.6	
	**Total (%)**	84.3	15.7		
		**Sleep efficiency**			
		Poor sleepers			
		**WH**			
		**0-1**	**2-3**	**Total (%)**	
	**0-1**	200	25	78.9	0.77
**Pre-WH**	**2-3**	22	38	21.1	
	**Total (%)**	77.9	22.1		
		Good sleepers			
		**WH**			
		**0-1**	**2-3**	**Total (%)**	
	**0-1**				<0.001[1]
**Pre-WH**	**2-3**				
	**Total (%)**				

**Sleep disturbances**					
Poor sleepers					
		**WH**			
		**0-1**	**2-3**	**Total (%)**	
	**0-1**	172	16	66.0	0.21
**Pre-WH**	**2-3**	25	72	34.0	
	**Total (%)**	69.1	30.9		
Good sleepers					
		**WH**			
		**0-1**	**2-3**	**Total (%)**	
	**0-1**	364	47	90.9	≪0.001
**Pre-WH**	**2-3**	6	35	9.1	
	**Total (%)**	81.9	18.1		
**Use of sleep medications**					
Poor sleepers					
		**WH**			
		**0-1**	**2-3**	**Total (%)**	
	**0-1**	231	11	84.9	1
**Pre-WH**	**2-3**	11	32	15.1	
	**Total (%)**	84.9	15.1		
Good sleepers					
		**WH**			
		**0-1**	**2-3**	**Total (%)**	
	**0-1**				0.03[1]
**Pre-WH**	**2-3**				
	**Total (%)**				
**Daytime dysfunction**					
Poor sleepers					
		**WH**			
		**0-1**	**2-3**	**Total (%)**	
	**0-1**	181	21	70.9	0.001
**Pre-WH**	**2-3**	49	34	29.1	
	**Total (%)**	80.7	19.3		
Good sleepers					
		**WH**			
		**0-1**	**2-3**	**Total (%)**	
	**0-1**	335	36	82.1	1
**Pre-WH**	**2-3**	36	45	17.9	
	**Total (%)**	82.1	17.9		

[1] Data not reported in compliance with the Italian legislation on the protection of personal data (Deontological rules for processing for statistical or scientific research purposes carried out within the National Statistical System**,** Resolution no. 514/2018).
